# The green ESCRTs: Newly defined roles for ESCRT proteins in plants

**DOI:** 10.1016/j.jbc.2025.108465

**Published:** 2025-03-27

**Authors:** Ethan Weiner, Elizabeth Berryman, Ariadna González Solís, Yuchen Shi, Marisa S. Otegui

**Affiliations:** Department of Botany and Center for Quantitative Cell Imaging, University of Wisconsin-Madison, Wisconsin, USA

**Keywords:** endosomes, membrane bending, intraluminal vesicles

## Abstract

Endocytosis and endosomal trafficking of plasma membrane proteins for degradation regulate cellular homeostasis and development. As part of these processes, ubiquitinated plasma membrane proteins (cargo) are recognized, clustered, and sorted into intraluminal vesicles of multivesicular endosomes by endosomal sorting complexes required for transport (ESCRT) proteins. At endosomes, ESCRT proteins recognize ubiquitinated cargo and mediate the deformation of the endosomal membrane in a negative geometry, away from the cytosol. ESCRTs are organized in five major complexes that are sequentially recruited to the endosomal membrane where they mediate its vesiculation and cargo sequestration. ESCRTs also participate in other membrane remodeling events and are widely conserved across organisms, both eukaryotes and prokaryotes. Plants contain both conserved and unique ESCRT components and show a general trend toward gene family expansion. Plant endosomes show a wide range of membrane budding patterns with potential implications in cargo sequestration efficiency, plant development, and hormone signaling. Understanding the diversification and specialization of plant ESCRT proteins can provide valuable insights in the mechanisms of ESCRT-mediated membrane bending. In this review, we discuss the endosomal function of ESCRT proteins, their unique features in plants, and the potential connections to the modes of plant endosomal vesiculation.

Endosomal sorting complexes required for transport (ESCRT) are a conserved group of proteins found in all kingdoms of life, from archaea to eukaryotes. As their name suggests, ESCRTs were first characterized as the machinery that bends the endosomal membrane to mediate vesiculation away from the cytosol (negative geometry), that is, in the opposite direction of other well-characterized vesiculation processes such as clathrin-mediated endocytosis. This means that, as ESCRTs are cytosolic proteins, they orchestrate membrane constriction from inside the vesicle neck (Fig. 1). This property of bending membranes in negative geometries was later found to be important for many other cellular processes, such as repair of the nuclear envelope (1, 2, 3, 4), plasma membrane, and lysosomal membranes (5, 6, 7), closure of autophagosomes during autophagy (8, 9, 10, 11, 12), cytokinesis abscission in metazoans (animals) (13, 14, 15, 16, 17, 18, 19), archaea (20, 21, 22, 23), and some unicellular red alga (24), nuclear envelope reformation after mitosis (25), formation of extracellular vesicles (26), shedding of damaged plasma membrane domains and viral budding in metazoan cells (27, 28, 29), and formation of intraluminal vesicles (ILVs) in plant peroxisomes (30) (Fig. 1). More recently, ESCRTs have been found to mediate membrane bending in positive geometries as well, such as during lipid droplet membrane remodeling to facilitate transfer of fatty acids to peroxisomes (31) and tubulation on chloroplastic membranes (32) (Fig. 1). Not surprisingly, due to their many central cellular roles, mutations in core ESCRT subunits of multicellular organisms lead to severe outcomes, including cancer and neurodegenerative diseases in mammals (33, 34, 35) and embryo or seedling lethality in plants (36, 37, 38).

**Figure 1:**
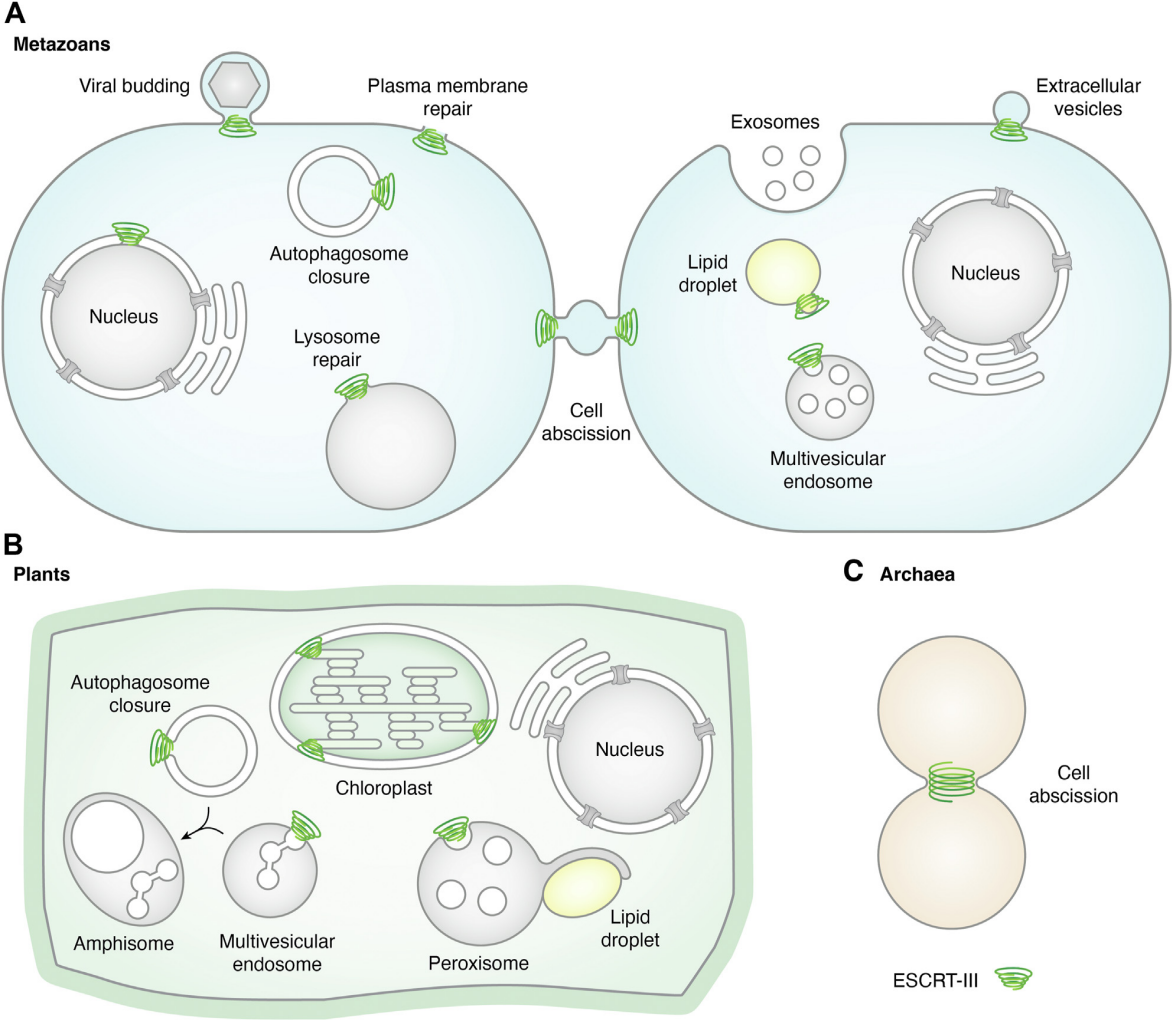
**ESCRT functions in cells.** Examples of cellular processes and organelles related to ESCRT functions in metazoans (*A*), Archaea (*B*), and plants (*C*). It is currently unclear whether some functions characterized in animal cells, such as repair/reformation of the nuclear envelope and lysosomal/vacuolar membrane repair, are conserved in plants. ESCRT, endosomal sorting complexes required for transport; LD, lipid droplets.

In their endosomal role, ESCRTs sort plasma membrane proteins (cargo) for vacuolar/lysosomal degradation. At the plasma membrane, ubiquitinated proteins targeted for degradation are internalized in vesicles *via* endocytosis, most commonly by clathrin-mediated endocytosis, and delivered to early endosomes. From there, cargo proteins are either recycled back to the plasma membrane or further transferred to late endosomes. At the endosomal limiting membrane, ESCRTs sort ubiquitinated cargo into ILVs that bud into the endosomal lumen, leading to the formation of multivesicular endosomes (MVEs), also called multivesicular bodies (Figure 1, Figure 2, Figure 3). MVEs ultimately fuse with lytic organelles (lysosomes or vacuoles) and release ILVs for their degradation. Through this mechanism, ESCRTs play a crucial role in the regulation of the plasma membrane composition.

**Figure 2:**
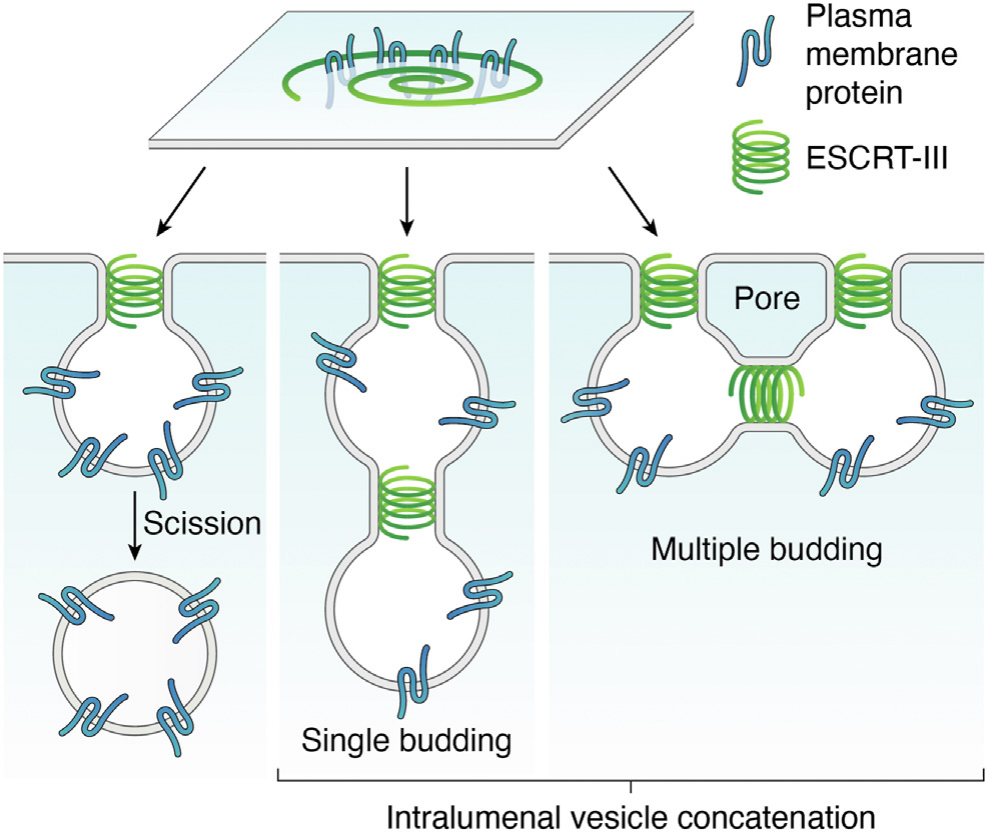
**ILV formation in eukaryotes**. ILVs in opisthokonts form by single budding followed by membrane scission whereas in plants, concatenated ILVs form as linear chains from single budding sites or from multiple budding sites that require membrane rupture and formation of pores (43). ILV, intraluminal vesicle.

**Figure 3:**
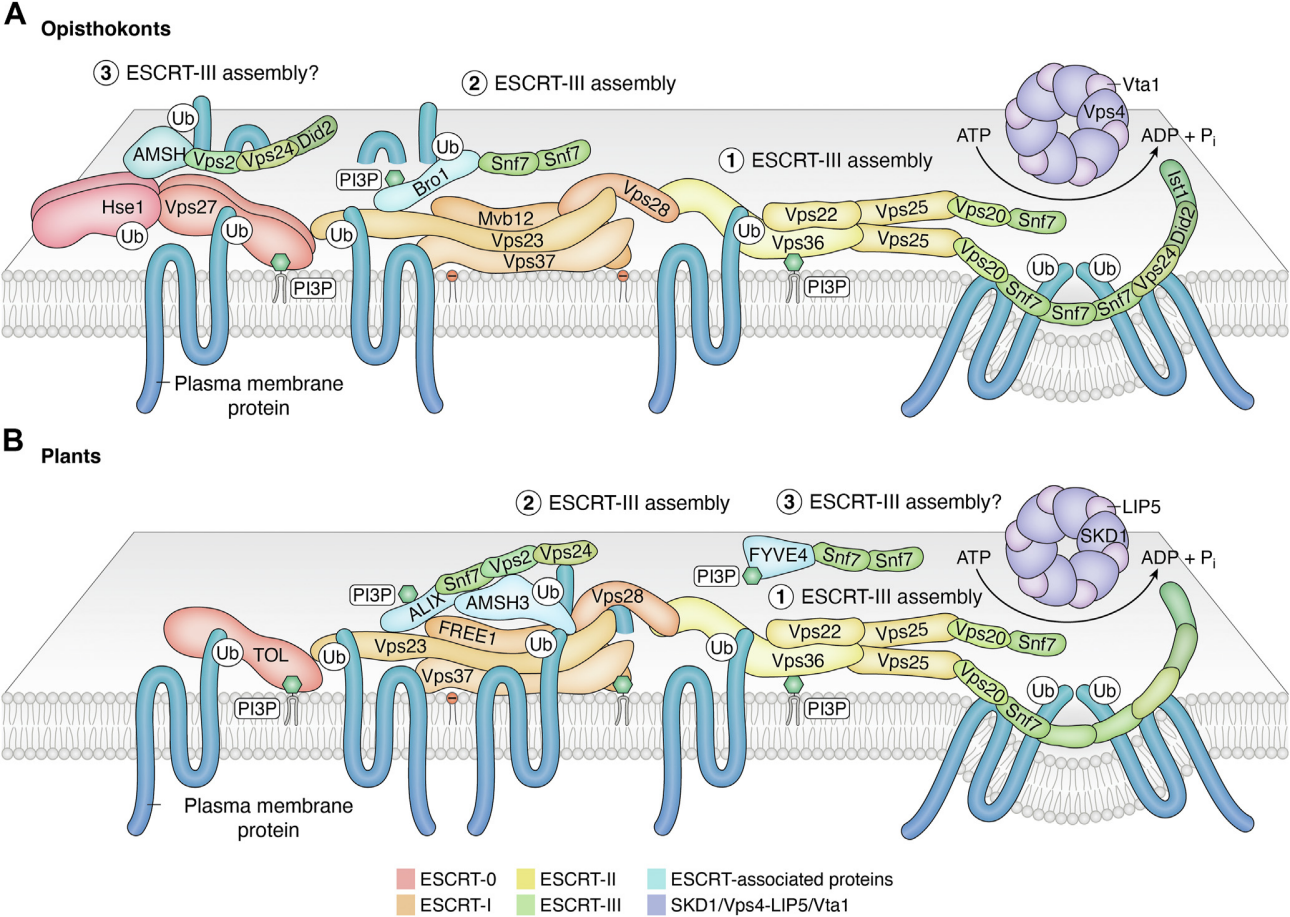
**Models of ESCRT organization on endosomal membranes in different organisms.** (*A*) Opisthokonts (yeast and animals) and (*B*) plants. The different ESCRT complexes are depicted in a sequential order but how exactly the different assemblies are organized with respect to each other and how dynamic their positions are during ILV formation are unknown. The two models include two pathways to initiate the assembly of the ESCRT-III filaments (ESCRT-III assembly site numbers 1 and 2), either through interactions with ALIX/Bro1 or with ESCRT-II. A third potential ESCRT recruitment (ESCRT-III assembly site number 3) could be coordinated by FYVE4 in plants and AMSH with Hse1/STAM of ESCRT-0 in opisthokonts. To facilitate visualization, the color scheme for the different ESCRT complexes matches the colors used in Figure 4. ALIX, Alg-2 interacting protein-X; AMSH, associated molecule with the SH3 domain of STAM; ESCRT, endosomal sorting complexes required for transport; ILV, intraluminal vesicle; STAM, signal transducing adaptor molecule.

Five main ESCRT assemblies, ESCRT-0, ESCRT-I, ESCRT-II, ESCRT-III, and the VPS4 (vacuolar protein sorting 4)-LIP5 (Lyst-interacting protein 5) complex, are recruited sequentially from the cytosol onto membranes (Figs. 3 and 4). ESCRT-0 proteins recognize plasma membrane cargo by binding ubiquitin moieties. ESCRT-0 recruits two more complexes with ubiquitin-binding capabilities, ESCRT-I and then ESCRT-II, the latter of which serves for nucleation of ESCRT-III polymers. ESCRT-III subunits polymerize into spiral filaments on the MVE membrane, which are remodeled through ESCRT-III subunit exchange and the action of the chaperone VPS4 (also known as SKD1 or suppressor of K^+^ transport growth defect 1 in plants) and its cofactor LIP5 (known as Vta1 or Vps twenty associated 1 in yeast). The remodeling of the ESCRT-III spirals facilitates the transition from flat (39, 40) to conical, and finally to a narrow cylindrical spiral that constricts the vesicle neck (41). In some organisms, membrane constriction leads to scission from the MVE limiting membrane, releasing the ILV into the lumen of the MVE, whereas in plants, constricted necks can be stable (42, 43) (Fig. 2).

**Figure 4:**
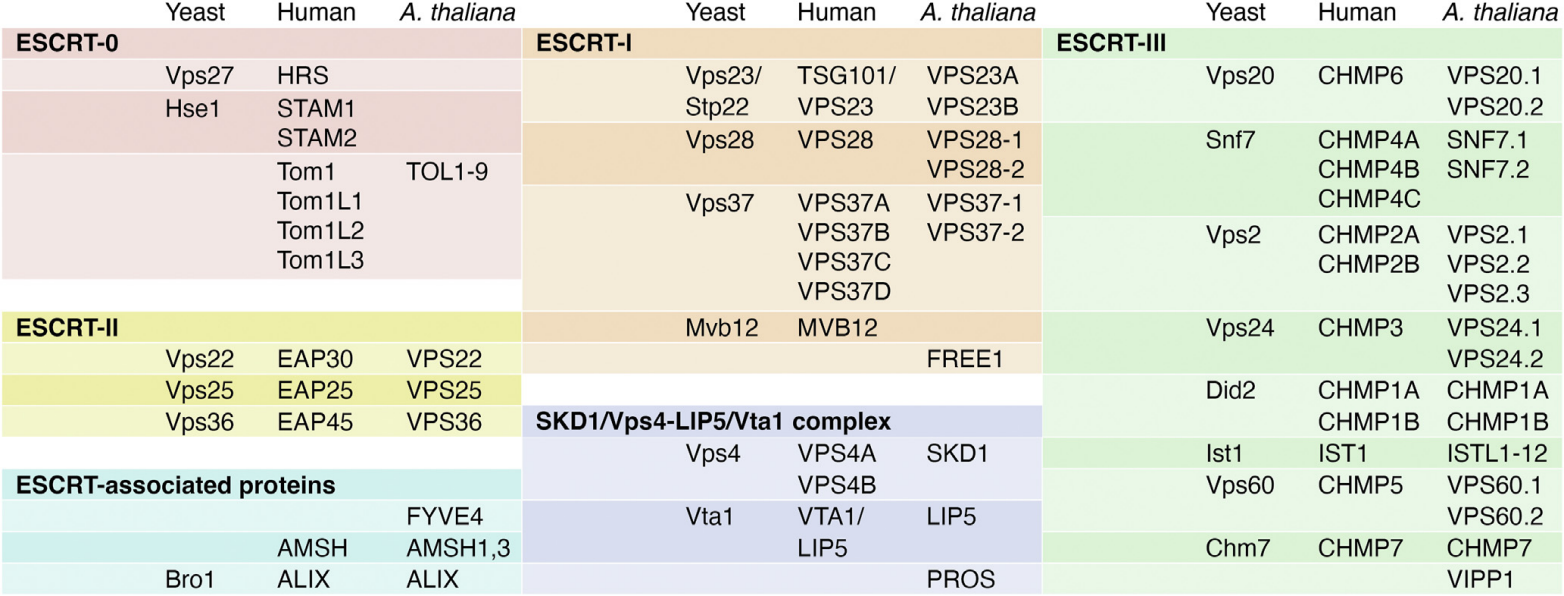
**ESCRT subunits in *Saccharomyces cerevisae* (budding yeast), humans, and *Arabidopsis thaliana***. ESCRT, endosomal sorting complexes required for transport.

Plants are a fascinating system to study this ancient family of proteins, as they have evolved some distinctive features. While the ESCRT proteins are in general well conserved across eukaryotes, plant ESCRTs show some notable peculiarities. For example, plants have unique ESCRT subunits (37, 44, 45) and numerous isoforms for some conserved ESCRT components (38, 46, 47, 48) (Fig. 4). Like other eukaryotes outside opisthokonts (animals and fungi), plants do not contain canonical ESCRT-0 proteins and instead have several TOM1-like (TOLs) proteins that play the role of ESCRT-0 (38).

Plant MVE architecture also differs from its fungal and animal counterparts; instead of releasing single ILVs at a time; plant MVEs can form complex concatenated networks of ILVs with limited membrane scission (Fig. 2) (42, 43). Therefore, while ILV neck constriction is coupled with membrane scission in yeast, it is not in plants. Plant concatenated ILVs form through either single budding sites that generate linear ILV chains or multiple budding sites that generate complex, interconnected ILV networks (Fig. 2). In both cases, ESCRT proteins remain associated with the ILV membranous necks with no evidence of recycling (42). Interestingly, these ILV budding patterns and the degree of ILV concatenation vary widely across plant lineages, with early divergent land plants characterized by low ILV concatenation and single budding and seed plants showing higher incidence of ILV concatenation and multiple budding sites (43). Multiple budding requires formation of a pore in the endosomal limiting membrane (Fig. 2) and, within seed plants, only occurs with high frequency in flowering plants (43). The biological significance of these changes in endosomal budding patterns across plant lineages is unclear, but one could speculate that the uncoupling of membrane constriction from scission and the capability to form several ILV simultaneously through multiple budding sites could increase cargo internalization and retention (42, 43).

Recent studies on the unique composition of the plant ESCRT assemblies and one in particular on endosomal budding patterns (43) have shed light on the mechanism by which the ESCRT machinery can bend membranes in both plants and other organisms. In this review, we will discuss the properties and functions of the different ESCRT assemblies, highlighting recent discoveries and plant-specific features.

## ESCRT-0

ESCRT-0 subunits are not well conserved across organisms. The best characterized ESCRT-0 complex consisting of the two subunits Vps27/HRS and Hse1/STAM is only present in opisthokonts (49, 50, 51, 52) (Figs. 3 and 4). These proteins assemble in a cytoplasmic heterotetrameric complex in a 2:2 ratio (53). Each possesses ubiquitin-binding domains that recognize ubiquitinated plasma membrane proteins targeted for degradation. HRS/Vps27 contains a VHS (Vps27/HRS/STAM) domain, a FYVE (Fab1b, YOTB, Vac1p, and EEA1) domain that binds phosphatidylinositol 3-phosphate (PI3P), a double ubiquitin-interacting motif consisting of an alpha helix able to interact with two ubiquitin molecules (54), a coiled-coil region that interacts with signal transducing adaptor molecule (STAM), and a clathrin-binding domain (55). STAM also contains a VHS domain and a ubiquitin-interacting motif, both of which can bind ubiquitin (56), and is capable of interacting with associated molecule with the SH3 domain of STAM (AMSH), which itself can bind ubiquitin. Thus, the STAM-AMSH complex can bind up to three ubiquitin molecules (57). HRS recruits the ESCRT-0 complex to early endosomal membranes through interaction of its FYVE domain with PI3P, a lipid enriched in endosomes (58), and it also interacts with the ESCRT-I component TSG101 (tumor susceptibility gene 101)/Vps23 (51).

Other eukaryotes do not have these ESCRT-0 subunits and instead use TOM1 (target of Myb1) protein family members, which bind both membranes and ubiquitin (59), for the initial steps in ESCRT recruitment (Figs. 3 and 4). Tom1 and Tom1L1, the mammalian homologues of TOM1, have N-terminal VHS domains like the canonical ESCRT-0 proteins and a central GAT (Golgi-localizing, γ-adaptin ear domain homology, ADP-ribosylation factor-binding protein and Tom1) domain, which binds ubiquitin (60). Both the VHS and GAT domains interact with negatively charged lipids, including PI3P (59). Just like HRS/Vps27, Tom1 also has a clathrin-binding domain (61).

There are nine TOL proteins in *Arabidopsis thaliana (Arabidopsis)* with partially redundant functions (38). Some of these have been experimentally shown to bind ubiquitin, act early in endosomal trafficking, and participate in sorting the auxin efflux carrier PIN2 (PIN-FORMED2) (38), a known plasma membrane ESCRT cargo. The *Arabidopsis* TOL proteins contain ubiquitin-binding VHS and GAT domains at their N terminus followed by clathrin-binding motifs (38). *Arabidopsis* TOL2 and TOL6, which localize to the cytoplasm and plasma membrane, respectively (38), interact with the ESCRT-I protein VPS23A, (62), supporting its role in recruiting downstream-acting ESCRT components. TOL2 and TOL6 bind preferentially to Lysine-63 (Lys-63)-linked ubiquitin. Mutations in the ubiquitin-binding sites abolish TOL6 plasma membrane localization and render it unfunctional (62). Interestingly, ubiquitination of TOL6 also increases the cytosolic localization while decreasing the functional pool at the plasma membrane (62). These findings support a regulatory mechanism for TOL function, whereby their ubiquitination state determines their subcellular localization and activity, thus controlling ESCRT activity overall and the turnover rate of cargo (62). Further evidence for the role of TOL proteins as early ESCRT components comes from another study that reports that a *tol2 3 5 6* quadruple mutant mis-sort abscisic acid (ABA) receptors (63), which are well-characterized ESCRT cargo (64, 65).

## ESCRT-I

In opisthokonts, ESCRT-I is a heterotetramer consisting of the subunits Vps23/TSG101, Vps28, Vps37, and Mvb12 (multivesicular body sorting factor of 12 kD) (66, 67, 68, 69, 70, 71) that coassembles on membranes with ESCRT-II in a super complex (72, 73) (Figs. 3 and 4). In mammals, an additional ESCRT-I subunit called UBAP1 (ubiquitin-associated protein 1) participates in the endosomal sorting of epidermal growth factor receptor; however, UBAP1 does not seem to be required for nonendosomal function such as cytokinesis (74). ESCRT-I is recruited to endosomes by interactions with acidic phospholipids (75) and with ESCRT-0 proteins (76). Structural studies in yeast showed that the ESCRT-I hetero-tetramer assembles into a rod-shaped complex. One end of the complex exposes the UEV (ubiquitin E2 variant) domain of Vps23 that interacts with ESCRT-0 and ubiquitin and the Vps37 basic N-terminal region that binds acidic phospholipids; at the other end, the C-terminal domain of Vps28 binds to the ESCRT-II subunit Vps36 (72). In animals, MVB12 has also been shown to interact with negatively charged phospholipids, anchoring the complex to the membrane (75).

Not all of the ESCRT-I subunits are conserved in plants; instead, plants contain a specific ESCRT-I subunit called FYVE1/FREE1(FYVE domain protein required for endosomal sorting 1), which is essential for plant development (37). The *Arabidopsis* genome encodes two homologues of each of the ESCRT-I subunits VPS23 (VPS23A and B), VPS28 (VPS28–1 and 2), and VPS37 (VPS37–1 and 2), but lacks MVB12 (Figs 3 and 4). The plant-specific FREE1 protein was first characterized for its ability to bind PI3P, localize at MVEs, and interact with VPS23 (37, 65, 77). FREE1 has three known domains: the N-terminal proline-rich intrinsically disordered region, the FYVE domain that binds PI3P, and a C-terminal coiled-coil region. FREE1 is incorporated in the ESCRT-I complex through its interaction with VPS23A and VPS23B (37). The intrinsically disordered region of FREE1 mediates its liquid-liquid phase separation and the formation of FREE1 condensates able to induce vesiculation in artificial membranes (78).

Both FREE1 and VPS23A have been shown to participate in the endosomal sorting of the ABA receptors, which control important pathways for plant growth, development, and stress responses. These two ESCRT-I components are required for the sorting of ubiquitinated pyrabactin resistance 1 (PYR1)/PYR1-like (PYL)/regulatory components of ABA receptors (RCAR)-type ABA receptors in MVEs for vacuolar degradation (65, 79). Consistent with this notion, the degradation of VPS23A and FREE1 by the proteasome reduces degradation of these receptors and enhances ABA signaling (79, 80). Interestingly, these ABA receptors lack transmembrane domains, representing one of the few examples of membrane peripheral proteins sorted for degradation by the ESCRT pathway. FREE1 also participates in the ABA signaling pathway by a different mechanism that involves recruitment to the nucleus. In response to ABA, phosphorylation of residues S530 and S533 at the C-terminal domain of FREE1 promotes its nuclear localization where it modulates the activation of ABA-responsive transcription factors (81).

In addition to its role in ABA signaling pathway, VPS23A is required for normal cytokinesis during trichome morphogenesis (82) and localization of SOS2 (salt overly sensitive 2) at the plasma membrane which activates SOS1, a Na+/H+ antiporter, to reduce the accumulation of sodium inside the cell (83). However, the cellular mechanistic bases for these VPS23A functions are not clear.

The two Arabidopsis VPS28 isoforms, VPS28-1 and VPS28-2, have been reported to be required for embryogenesis and auxin distribution during development, likely through the abnormal trafficking of plasma membrane auxin carriers (84).

In the last decade, the study of ESCRT-I proteins in plants has uncovered novel functions beyond their role in endosomal sorting. For example, FREE1 also has a role in the degradation of lipid droplets during seed germination, where the interaction of FREE1 with the peroxisomal protein PEX11e (peroxin 11e) and the lipase SDP1 (sugar-dependent protein 1) regulates peroxisomal extension and tubulation to facilitate the engulfment of lipid droplets by peroxisomes and the degradation of triacylglycerols (85) (Fig. 1). FREE1, as other ESCRT subunits, also participates in the catabolic process of autophagy, in which portions of cytoplasm, organelles and/or protein aggregates are surrounded by a double membrane structure called an autophagosome and delivered to vacuoles for degradation (Fig. 1). For example, FREE1 interacts with SH3P2 (SH3 domain-containing protein 2), a key regulator of plant autophagy (86), and *FREE1*-knockdown plants accumulate aberrant autophagosomes (86). In a more recent study, FREE1 was shown to play a role in autophagosome closure in a nutrient-dependent manner (12). Under nutrient starvation, the energy-sensitive kinase SnRK1α1 (sucrose nonfermenting 1-related kinase 1 α1) phosphorylates FREE1, promoting its interaction with ESCRT-III subunits, the autophagy ATG12-ATG5-ATG16 conjugation machinery, and the conjugation substrate ATG8 to facilitate the closing of autophagosomes (12).

In animal cells, the fusion between endosomes and autophagosomes generates amphisomes, which mature into lytic autolysosomes (87). A similar process facilitated by ESCRT-I has been recently reported in plant cells, where VPS23A interacts with the autophagy adaptor CFS1 (cell death related endosomal FYVE/SYLF protein 1) to tether MVEs and autophagosomes and facilitate the formation of amphisomes (Fig. 1) that deliver both endosomal and autophagic cargo to the vacuole (88).

## ESCRT-II

ESCRT-II is a Y-shaped heterodimer consisting of three subunits, Vps22/EAP30, Vps25/EAP20, and Vps36/EAP45, in a 1:2:1 stoichiometry (89) (Figs. 3 and 4). The three ESCRT-II subunits are paralogs that share the presence of two WH (winged helix) domains (90). Vps22 and Vps36 form the core of the complex whereas the two Vps25 proteins form the arms of the Y (91, 92, 93). Just like ESCRT-0, TOL proteins, and ESCRT-I, ESCRT-II binds PI3P and ubiquitin. Vps36 contains a GLUE (gram-like ubiquitin-binding in EAP45) domain that binds membranes and ubiquitin (94, 95, 96) whereas Vps25 is critical for the recruitment of downstream ESCRT components through binding to the ESCRT-III subunit Vps20 (97).

Plants seem to contain conserved ESCRT-II subunits (Figs. 3 and 4). However, this is the least studied plant ESCRT complex, and it has not been characterized biochemically. There is only one VPS36 gene in *Arabidopsis*, which is essential for normal embryo development and seedling survival (98). Similar to other core ESCRT mutants, *vps36* mutant seedlings mislocalize their plasma membrane influx and efflux auxin carriers, explaining their severe developmental defects (98). Mutant seeds for *VPS22* in rice (*Oryza sativa*) showed a chalky and abnormal endosperm and develop into abnormal seedlings that die a few days after germination (99).

## ESCRT-III

While ESCRT-0, TOL, ESCRT-I, and ESCRT-II components function in ubiquitinated cargo recognition and clustering at the MVE surface, the ESCRT-III proteins and Vps4 are the ultimate drivers of membrane bending and vesicle formation. In fact, these proteins alone are sufficient to induce *in vitro* membrane deformation in giant unilamellar vesicles (100, 101).

There are eight conserved core ESCRT-III proteins in opisthokonts and plants: CHMP2 (charged multivesicular body protein 2)/Vps2, CHMP4/Snf7, CHMP3/Vps24, CHMP6/Vps20, CHMP1/Did2, CHMP5/Vps60, Ist1, and CHMP7 (Figs. 3 and 4). At least in metazoans, CHMP7 does not seem to function in endosomal sorting but instead in repairing the nuclear envelope (102, 103). ESCRT-III proteins are characterized by a conserved N-terminal coiled-coil domain of multiple alpha helices (104, 105) with one or more MIT-interacting motif (MIM) domains, which allow them to interact with the microtubule-interacting and trafficking (MIT) domains of other proteins, such as Vps4 and LIP5 (106, 107). ESCRT-III proteins form helical, single or double-stranded heteropolymers and deform membranes. ESCRT-III polymerization is regulated by their C-terminal autoinhibitory domain which acts like a hinge that opens and closes to allow or prevent association with membranes and other ESCRT-III monomers, respectively (104).

ESCRT-III polymerization can be initiated by interactions with a few different components of the ESCRT machinery (Fig. 2). For example, the ESCRT-II subunit Vps25 recruits Vps20 (90, 108) (Fig. 3; ESCRT-III assembly site 1). This is followed by the incorporation of other ESCRT-III subunits, which assemble into filamentous copolymers (41, 109, 110, 111). Based on the analysis of ESCRT-III subunits, a model has been proposed where the sequential polymerization and exchange of Snf7, Vps2, Vps24, Did2, and Ist1, facilitated by the chaperone Vps4, change the shape of the ESCRT-III assembly from a flat spiral to a constricted, cylindrical spiral, driving membrane deformation and scission (41, 109). The ESCRT-III protein Vps60 can also polymerize into spiral filaments and copolymerize with other ESCRT-III subunits *in vitro* (109, 112), but its specific role in membrane deformation is unclear. It is also unclear whether the models based on *in vitro* studies reflect the dynamic behavior of ESCRT-III proteins at the endosomes, where other proteins and specific membrane lipid compositions could alter their conformation and dynamics.

The ESCRT-III components Did2/CHMP1, Ist1, and Vps60 have been shown to stimulate VPS4 ATPase activity either through direct interaction or *via* its cofactor LIP5/Vta1 (113). It is not completely clear how ESCRT-III proteins function. While they are recruited sequentially from the cytosol to deform the endosomal membrane (41), there is evidence that they can exist in different functional states, with some promoting assembly and ILV formation and others, complex disassembly (114).

Within the ESCRT-III subunits, Ist1 has some unique structural and functional features. It does not seem to be required for MVE vesiculation in yeast (111, 115, 116) and plants (47) but it does participate in ILV formation in *Caenorhabditis elegans* (117). It has also been shown to mediate endosomal recycling in animal cells (118, 119) in connection to its capacity to bend membrane toward the cytosol (positive geometry). Ist1 has two MIM domains (instead of just one like other ESCRT-III subunits) and copolymerizes with CHMP1 forming filaments that constrict membranes from outside (instead of inside) (120, 121, 122), which leads to the tubulation and scission of recycling endosomal membranes in animal cells (119). Recent cryo-electron microscopy studies of CHMP1-IST1 copolymers on labeled lipid membranes have shown that CHMP1 residues can change the organization of the membrane leaflet they contact and stabilize membrane curvature (123).

ESCRT-associated proteins also contribute to local membrane remodeling needed for construction and scission. For example, the Vps60-interacting protein Vps68 has been postulated to facilitate ILV scission in yeast by weakening the cytosolic leaflet of the membrane at the ILV neck (114).

All eight conserved core ESCRT-III subunits are present in plants, although most of them are represented by several isoforms (Fig. 4). Consistent with their role in endosomal sorting and other specialized cellular functions, mutations in core ESCRT-III and ESCRT-III associated proteins in plants result in abnormal endosomes with reduced number of ILV and ILV concatenation and serious developmental defects, embryo lethality, and/or abnormal responses to biotic and abiotic stress (36, 47, 124, 125, 126, 127, 128, 129, 130, 131).

The underlying mechanism for multiple ILV budding in seed plants is unknown, but molecular simulations predict that it could be connected to changes in the curvature and/or membrane binding energy of the plant ESCRT-III filaments (43). Thus, one possibility is that the broad expansion of plant ESCRT-III subunits was accompanied by changes in their biophysical and structural properties and ultimately, in novel ILV budding patterns. For example, whereas most ESCRT-III subunits are represented by one to three isoforms in yeast and humans, all ESCRT-III subunits have multiple isoforms in *Arabidopsis*. In an extreme case, the *Arabidopsis* genome contains 12 IST1-like (ISTL) genes (Fig. 4). Interestingly, Ist1 is thought to mediate the last step in membrane constriction (41), opening the possibility that some of the plant ISTL subunits could be responsible for the unique endosomal budding patterns in plant MVEs. However, based on mutant analysis and protein-protein interaction assays, not all *Arabidopsis* ISTL proteins seem to have conserved endosomal functions (47, 124). More structural and genetic work will be needed to determine the function of the ISTL protein family in plants.

## ESCRT-III-related proteins

ESCRT-III proteins can also be recruited by other ESCRT-related proteins, including ALIX (Alg-2 interacting protein-X)/BRO1, the AMSH deubiquitinases, and FYVE4. ALIX/BRO1 is conserved in eukaryotes and contains a C-terminal proline-rich domain that interacts with the ESCRT-I subunit TSG101 (132), a middle V-shaped domain (V-domain) that binds ubiquitinated protein cargo (133), and an N-terminal Bro1 domain that binds the ESCRT-III subunit Snf7 (134), initiating ESCRT-III assembly (Fig. 3, ESCRT-III assembly site 2). Thus, it has been postulated that ALIX and the ESCRT-III subunit Vps20 may be acting in parallel pathways to promote the recruitment and oligomerization of Snf7 filaments on membranes (135) (Fig. 3). The proline-rich domain of ALIX mediates its reversible tyrosine phosphorylation-dependent phase-separation. ALIX condensates sequester the human Snf7 paralogues CHMP4B and CHMP4C involved in cytokinesis and ALIX condensation regulates the timing and progression of cell abscission (136). In addition, human ALIX interacts with the autophagic proteins ATG12 and ATG13 and is required for basal autophagic activities (137).

In *Arabidopsis*, ALIX interacts with the ESCRT components FREE1, VPS23/TSG101, and SNF7 (138, 139). ALIX participates in endosomal cargo sorting and interacts with the PYR/PYL ABA receptors, mediating their degradation. By controlling their degradation *via* endosomal trafficking, ALIX is a negative regulator of ABA-dependent cellular responses, including stomatal closure (64). Plant ALIX also recruits ESCRT components to autophagic membranes for autophagosome closure (Fig. 1). The mechanism by which ALIX facilitates autophagosome formation has been recently elucidated. ALIX interacts with a Ca^2+^-dependent lipid binding protein 1 (CaLB1) and both bind PI3P, a lipid enriched in autophagosomal membranes, and both undergo phase separation. CaLB1 also interacts with ATG8, which decorates autophagic membranes, directing the localization of ESCRT proteins on autophagosomes, especially during salt-induced autophagy (10).

Plant ALIX also recruits AMSH3 to endosomes (129). AMSH proteins are deubiquitinases with specificity for Lys63-and Lys48-linked polyubiquitin chains (140, 141, 142) that contain an MIT domain and may be important for disengaging ubiquitin chains from ESCRT components or deubiquitinating cargo. *Arabidopsis* mutants for AMSH3 show impaired endocytosis and vacuolar trafficking as well as accumulation of autophagosomes (130). Similar to what has been reported for human AMSH (143, 144), the MIT of AMSH3 competes with SKD1/Vps4 for binding the MIM domains of the ESCRT-III proteins, such as VPS2.1 and VPS24 (128). Whereas human AMSH binds the ESCRT-0 subunit STAM, AMSH3 binds the ESCRT-I component VPS23A (127), suggesting that both human and plant AMSH proteins could be supporting nucleation of ESCRT-III filaments but from different complexes: ESCRT-I-ALIX in plants and ESCRT-0 in humans (Fig. 3).

Finally, FYVE4 is a plant-specific FYVE-domain containing protein that binds phosphoinositides. While FYVE domains in other ESCRT components such as FREE1 and Vps27 facilitate membrane recruitment of early acting protein complexes (37, 50), *Arabidopsis* FYVE4 surprisingly binds ESCRT-III component SNF7, but not ESCRT-I, -II, or other ESCRT-III proteins in yeast-two hybrid assays. Further, FYVE4 was shown to be important for SNF7/ESCRT-III recruitment to the endosomes and for ILV formation (45). However, whether FYVE4 represents an independent pathway to recruit ESCRT-III polymers to endosomes remains unclear (Fig. 3).

In addition to homologues for the canonical ESCRT-III subunits, plants also contain vesicle-inducing protein in plastids 1 (VIPP1), a bacterial, chloroplastic ESCRT-III-like protein. VIPP1 is required for thylakoid membrane biogenesis (Fig. 1) and remodeling under stress in plants (145), green algae (*e.g. Chlamydomonas reinhardtii* (146)), and cyanobacteria (147). VIPP1 contains six or seven alpha helices, with the N-terminal one mediating binding to lipids (32), membrane deformation (148), and GTP/ATP hydrolytic activity (149). *Chlamydomonas* VIPP1 assembles into hollow rods that engulf and tubulate liposomal membranes (150), further confirming the membrane remodeling capabilities of this protein. Interestingly and different from the eukaryotic ESCRT-III complex, VIPP1 is the only cyanobacterial/chloroplast ESCRT-III subunit, opening many questions on its assembly and constriction mechanism. Recent studies have shown that VIPP1 forms flexible filaments of variable thickness that can form spirals and rings, but also, and different from other ESCRT-III proteins, planar polygonal arrays (148, 151, 152, 153). The VIPP arrays can transition from planar to three-dimensional architectures facilitating membrane bending.

## The VPS4-LIP5 complex

The last steps in ESCRT-mediated membrane bending involves the participation of the chaperone VPS4 (SKD1 in plants) and its cofactor LIP5 (Vta1 in yeast), which remodel the ESCRT-III polymers and drive membrane vesiculation in an ATPase-dependent manner (39, 41, 101) (Fig. 3). VPS4 is a AAA (ATPases associated with diverse cellular activities) ATPase with an MIT domain that interacts with the ∼20-residue MIM domains of ESCRT-III proteins (106), an ATPase cassette composed of the large and small ATPase domains that hydrolyze ATP (154), and a β domain that binds LIP5 (155). Like other AAA ATPases, VPS4 cycles between inactive and active states. In yeast, the inactive form of Vps4 has been proposed to exist as dimer (156) or a monomer (157) while the active state is a hexamer (155, 158) or a dodecamer (156, 157). The functional oligomeric form of Vps4 is stabilized by the binding of Vta1 dimers, which form bridges between adjacent Vps4 subunits (113, 159). The active hexameric Vps4 bound to Vta1 adopts an asymmetric helical configuration (155, 159), composed of monomers in different energy states, supporting a model in which the ATP-bound state promotes the growth of the Vps4 helix at one end while ATP hydrolysis promotes disassembly at the other end, allowing the Vps4 hexamer to “walk” along ESCRT-III filament while removing subunits through the central Vps4 pore (159). Through this process, Vps4 is thought to mediate the remodeling of the ESCRT-III assemblies and exchange of subunits (41, 160, 161). VPS4 is also believed to mediate membrane scission and vesicle release in Opistokhonts, but that does not seem to be the case in plant endosomes, in which vesicle necks remain stable and membrane constriction and scission are uncoupled (Fig. 3) (42, 43).

LIP5 has a dual function by acting as a strong activator of VPS4 ATPase activity (162, 163, 164) and recruiting ESCRT-III components. Accordingly, LIP5 contains a well conserved C-terminal domain called VSL (for Vta1 SBP1 and LIP5) that mediates both LIP5 dimerization and Vps4 binding (162, 165) and an N-terminal region with two MIT domains (166) that interacts with the ESCRT-III subunits Ist1 (18), CHMP1/Did2 (164), and Vps60 (167). LIP5 recruits Vps60 to endosomal membranes and mutations in both LIP5 and Ist1 lead to synergistic trafficking defects in yeast (111, 115), mammalian cells (18), and plants (47, 124). However, mutations in LIP5 results in much less severe mis-sorting defects than those in Vps4 (47, 163, 168), suggesting a nonessential role of LIP5 in supporting VPS4 function.

*In vitro*, yeast VPS4 and the ESCRT-III subunits Snf7, Vps2, and Vps24 are enough to mediate membrane scission (101). However, whereas VPS4 is needed to remodel ESCRT-III assemblies that lead to membrane constriction, it may not directly mediate membrane scission in all organisms and ESCRT-dependent processes. As mentioned, plants developed endosomal ILVs interconnected by ESCRT-III-decorated membranous bridges, supporting the idea that membrane constriction and scission can be uncoupled (42). Whether this is due to unique properties of plant ESCRT-III subunits or the plant VPS4 counterpart, SKD1, is unclear.

SKD1 is an essential gene in plants (163, 169) and its interaction with LIP5 is conserved (163). Consistent with results in yeast, plant LIP5 is not essential for SKD1 function, as the *lip5* mutant is viable and shows relatively mild phenotypic defects connected to abnormal auxin transport (47) and enhanced sensitivity to heat, high salt, and pathogen infection (170, 171). In addition, whereas *Arabidopsis* mutants for ISTL1 (the *Arabidopsis* Ist1 homologue) show no detectable defects, the deletion of both *LIP5* and *ISTL1* lead to synergistic cellular defects and ultimately to plant death (47). However, the *istl1 lip5* double mutant shows additional trafficking defects not directly connected to endosomal trafficking. Indeed, secretion of both plasma membrane proteins and extracellular exocytic cargo is affected in tapetal cells needed for pollen formation (124). These results cannot be easily explained by the role of LIP5 and ISTL1 in endosomal sorting and may be connected to the divergent functions of the Ist1 subunit, which in animals and in combination with CHMP1, also mediates positive membrane deformation (120, 122) and protein cargo recycling from early endosomes (118, 119).

In addition to LIP5, plants have a specific SKD1 regulator, PROS (positive regulator of SKD1), which enhances SKD1 ATPase activity *in vitro* and promotes cell expansion and organ growth (44). The plant SKD1-LIP5 complex represents a promising area for future study due to its central importance in regulation of ESCRT mediated membrane deformation and one potential cause for the unique endosomal budding patterns seen in plants.

## Conclusions and open questions

A wealth of new information on ESCRT function is coming from structural and comparative studies across highly divergent organisms. Recent structural studies of ESCRT complexes, especially of ESCRT-III assemblies, are revealing the underlying biophysical mechanisms by which ESCRT machinery interacts with membranes and mediates membrane remodeling. The plasticity of the ESCRT arrays has also been revealed by the discovery that IST1-CHMP1 copolymers can bend membranes in positive geometries (120). The comparative analysis of ESCRT proteins across organisms, including Archaea and plants, has uncovered variations in the mechanisms by which this ancestral molecular machinery has acquires its capabilities to bend membranes in the context of a multitude of cellular processes. In addition, the finding that the phase separation and condensation of the plant specific ESCRT-I subunit FREE1 is required for membrane vesiculation (78) opens many questions as to whether other organisms require phase separation of ESCRT components to mediate ILV formation and which ESCRT subunits beside FREE1 and ALIX may show similar behavior.

Recent electron tomography studies have shown that MVEs differ greatly in morphology and budding mechanisms across plant lineages (43). This type of analysis allows for testing hypotheses on what unique features of ESCRT proteins (*e.g.* membrane binding energy, filament curvature) have evolved within plants. However, it is quite possible that comparable morphological differences in MVE structure and budding exist across eukaryotes, providing exciting opportunities for studying ESCRT-mediated membrane bending in organisms outside opisthokonts.

## Conflict of interest

The authors declare that they have no conflicts of interest with the contents of this article.
